# Isolated T Wave Inversion in Lead aVL: An ECG Survey and a Case Report

**DOI:** 10.1155/2015/250614

**Published:** 2015-04-09

**Authors:** Getaw Worku Hassen, Ana Costea, Claire Carrazco, Tsion Frew, Anand Swaminathan, Jason Feliberti, Roger Chirurgi, Tennyson Smith, Alice Chen, Sarah Thompson, Neola Gushway-Henry, Bonnie Simmons, George Fernaine, Hossein Kalantari, Soheila Talebi

**Affiliations:** ^1^Lutheran Medical Center, Department of Emergency Medicine, Brooklyn, NY 11220, USA; ^2^NYMC, Metropolitan Hospital Center, Department of Emergency Medicine, New York, NY 10029, USA; ^3^Department of Emergency Medicine, St. George's University School of Medicine, St. George's, Grenada; ^4^School of Osteopathic Medicine, A.T. Still University, Mesa, AZ 85206, USA; ^5^Bellevue Medical Center, Department of Emergency Medicine, New York, NY 10016, USA; ^6^Department of Internal Medicine, NYU, New York, NY 10016, USA; ^7^Medgar Evers College, Brooklyn, NY 11225, USA; ^8^Lutheran Medical Center, Department of Family Practice, Brooklyn, NY 11220, USA; ^9^Lutheran Medical Center, Department of Internal Medicine, Brooklyn, NY 11220, USA; ^10^NYMC, Metropolitan Hospital Center, Department of Internal Medicine, New York, NY 10029, USA

## Abstract

*Background.* Computerized electrocardiogram (ECG) analysis has been of tremendous help for noncardiologists, but can we rely on it? The importance of ST depression and T wave inversions in lead aVL has not been emphasized and not well recognized across all specialties.* Objective.* This study's goal was to analyze if there is a discrepancy of interpretation by physicians from different specialties and a computer-generated ECG reading in regard to a TWI in lead aVL.* Methods.* In this multidisciplinary prospective study, a single ECG with isolated TWI in lead aVL that was interpreted by the computer as normal was given to all participants to interpret in writing. The readings by all physicians were compared by level of education and by specialty to one another and to the computer interpretation.* Results.* A total of 191 physicians participated in the study. Of the 191 physicians 48 (25.1%) identified and 143 (74.9%) did not identify the isolated TWI in lead aVL.* Conclusion.* Our study demonstrated that 74.9% did not recognize the abnormality. New and subtle ECG findings should be emphasized in their training so as not to miss significant findings that could cause morbidity and mortality.

## 1. Introduction

The electrocardiogram (ECG) is the most important initial diagnostic tool for myocardial infarction (MI). The computer is helpful in accurately identifying common pathologies on ECG. Changes in certain leads represent possible myocardial injury in specific coronary artery distributions. Therefore MI location could be inferred based on the area affected within the ECG [1, 2]. ECG changes of myocardial injury and ischemia include hyperacute T wave, ST elevation, Q wave, ST depression, T wave flattening as well as T wave inversion (TWI) [2, 3]. ST elevation has been recognized as a marker for acute myocardial infarction. ST depression and TWI are the result of myocardial ischemia except in leads V1 and V2, which may represent posterior myocardial infarction [3, 4].

Reciprocal changes such as ST depressions and TWI are well-recognized ECG changes that accompany ST elevations [5, 6] and their significance has been the subject of many studies. These changes have been studied to localize the site of a coronary lesion [7] and also are believed to be early and sensitive markers of myocardial infarction [8]. Reciprocal changes may be the only manifestation of acute myocardial ischemia and may be present in a single lead as ST depression in lead aVL [5, 6] and TWI in lead aVL indicating a significant coronary artery lesion in left anterior descending (LAD) artery is another example [9–11]. The importance of these changes has not been emphasized and/or well recognized across all specialties.

The objective of this study was to determine the ability of physicians to identify isolated TWI in lead aVL on ECG that is read as normal by ECG computer. In addition, we sought to analyze if there is a discrepancy in interpretation in regard to a TWI in lead aVL by physicians from different specialties and a computer-generated ECG. To the best of our knowledge no such study was conducted previously.

## 2. Material and Methods

This prospective study was multicenter and multidisciplinary. The participating physicians were from the departments of Emergency Medicine (EM), Family Practice (FP), Internal Medicine (IM), and Surgery (S) of four different hospitals. These physicians had different training levels (attending physician and resident physicians of different postgraduate levels). They were grouped according to their training level: postgraduate year (PGY1–5) and attending physicians of each specialty. Institutional review boards (IRBs) of the corresponding institutions approved the study independently.

**Figure 1:**
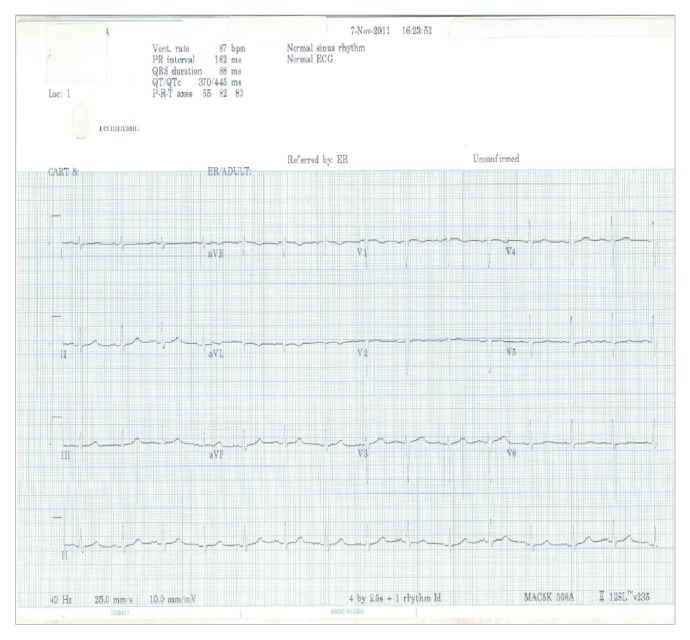
ECG representing isolated T wave inversion in lead aVL.

A single ECG with the isolated TWI in lead aVL that was interpreted by the computer as normal was given to all participants with a case description (Figure 1). Physicians were not allowed to consult with one other and they were given 5 minutes to perform their reading. All physicians gave their interpretations in writing after reviewing the case presentation and the corresponding ECG. The readings by all physicians and the computer interpretation were compared in regard to changes in lead aVL as well as amongst all physicians by level of education and by specialty.

### 2.1. Statistical Methods and Results

Using generalized linear models in the statistical programming software R, a forward stepwise logistic regression analysis was performed.

## 3. Results

**Figure 2:**
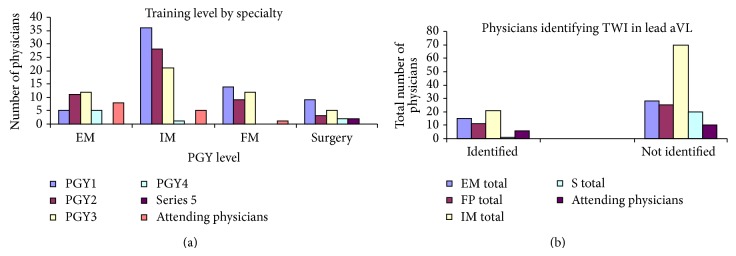
(a) Number of physicians by training level and specialties. (b) Total number of physicians who identified TWI in lead aVL on ECG by training level and specialties.

A total of 191 physicians participated in the study. Of the 191 physicians 43 (22.5%) were EM physicians, 91 (58.1%) were IM physicians, 36 (18.8%) were FP physicians, and 21 (11%) were surgeons (Table 1). In terms of training level, 64 (33.5%) physicians were PGY1, 51 (26.7%) physicians were PGY2, 50 (26.2%) physicians were PGY3, 8 (4.2%) physicians were PGY4, 2 (1%) physicians were PGY5, and 16 (8.4%) physicians were attending physicians. A total of 48 (25.1%) physicians identified and 143 (74.9%) physicians did not identify the isolated TWI in lead aVL. Of the 48 physicians that identified the isolated TWI in lead aVL 15 (31.3%) were EM physicians and 21 (43.8%) were IM physicians, 11 (23%) were FP physicians, and 1 (2%) physician was surgeon. In terms of training level of physicians who identified the isolated TWI in lead aVL, 13 (27.1%) were PGY1, 16 (33.3%) were PGY2, 12 (25%) were PGY3, 2 (4.2%) were PGY4, and 6 (12.5%) were attending physicians (Table 1 and Figures 2(a) and 2(b)). Using generalized linear models in the statistical programming software R, a forward stepwise logistic regression analysis was performed. Year of training was more significant than specialty in terms of predicting the odds of identifying TWI in lead aVL. Emergency Medicine physicians overall had the highest odds, while surgeons had the lowest odds, of identifying TWI in lead aVL. Controlling for specialty, there is an increase in odds of identification for both PGY2 and PGY3 physicians and a decrease in the odds of identification for both PGY4 and PGY5 physicians. Attending physicians have an increase in the odds of identification.

## 4. Case Report

This is the case of a 69-year-old man who presented to the emergency department (ED) with left sided chest pain for 3 days. The pain was sudden in onset, sharp in nature, and 5-6/10 in severity. He noticed the pain while walking and it radiated to the left shoulder. He took aspirin and his pain improved to 1/10, but he continued to have dull substernal chest pain without radiation. This pain was exacerbated by deep palpation and cold weather. He denied shortness of breath, dizziness, fever, chills, nausea, or vomiting. He was seen approximately 6 months ago for a nuclear stress test, which was negative. His ECG in September 2011 was sinus rhythm at 62 beats per minute, normal axis, and Q wave in lead III and upright T wave in lead aVL.

His past medical and surgical history included hypertension, benign prostate hyperplasia, gastroesophageal reflux disease (GERD), and surgery for colon cancer. He denied using alcohol or tobacco.

**Figure 3:**
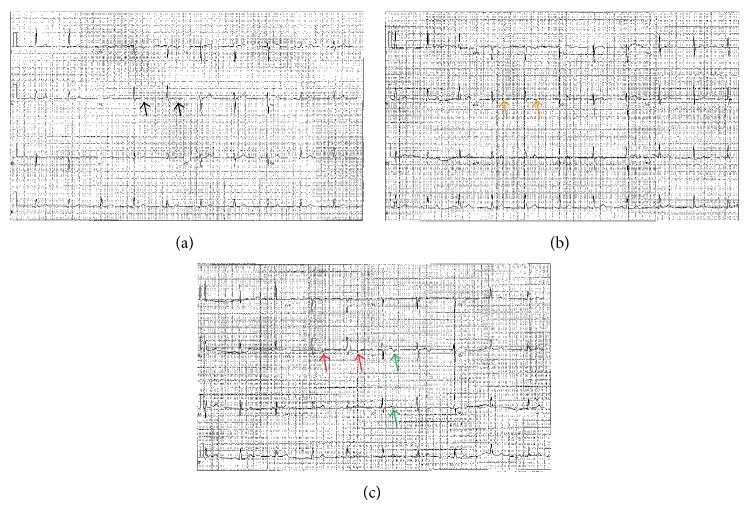
Dynamic T wave changes on ECG. (a) Upright T wave in lead aVL (black arrows, old ECG); (b) T wave flattening and early inversion in lead aVL (orange arrows, ECG at presentation); (c) TWI in lead aVL (red arrows, repeat ECG) and biphasic T waves in leads V2 and V3 (green arrows).

**Figure 4:**
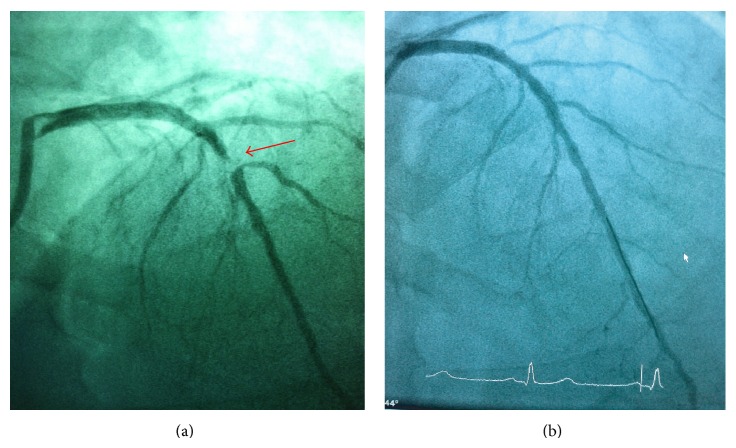
Coronary angiography. (a) Mid-LAD lesion (red arrow) and no proximal LAD abnormality: (b) after placement of stent.

**Figure 5:**
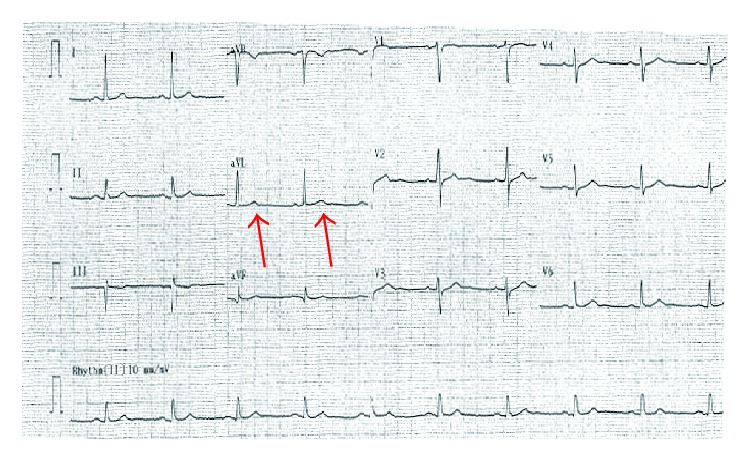
Follow-up ECG: patient returned to the cardiology office and his repeat ECG demonstrated the upright T waves in lead aVL after the stent placement.

On presentation his vital signs were as follows: temperature 98.4 degrees Fahrenheit, respiration rate 18 breaths per minute, pulse 82 beats per minute, and blood pressure 145/80 mmHG. His physical examination was unremarkable except for mild sternal tenderness to palpation. His initial ECG showed sinus rhythm at 64 and T wave flattening in lead aVL (Figure 3(b)). The chest radiography was unremarkable. The repeat ECG showed sinus rhythm at 62 beats per minute with TWI in lead aVL along with biphasic T waves in leads V2 and V3 (Figure 3(c)). A comparative old ECG has normal T wave in lead aVL (Figure 3(a)). The patient was taken to the angiography suite and underwent a coronary angiogram. Angiography revealed a 99% mid-LAD lesion and he received a stent (Figures 4(a) and 4(b)). Laboratory results were unremarkable except for mildly elevated triglyceride levels (202 mg/dL) and white blood cell count (15, 600 mm^3^). Cardiac troponins were within normal limits. The patient was subsequently discharged home to follow up as an outpatient. He remained symptom-free and on the follow-up clinic visit his ECG normalized with upright T waves in lead aVL (Figure 5).

## 5. Discussion

Computerized ECG analysis has been one of the most rapidly and widely adopted computer applications in medicine. A computer-assisted interpretation could be valuable especially to the noncardiologist physician [12]. It was pioneered in the 1960s and 1970s [13–15]. A system developed by Pipberger et al. was capable of automatic recognition of electrocardiographic waves by digital computer [14–16]. Two principles were used for automated ECG analysis. The first system involves pattern recognition techniques of ECG signals that have been previously recorded and stored in digital computer [16, 17]. Another program applied decision tree logic to measurements of waveform amplitude and duration [12]. Second generation programs were designed that employed statistical methods for diagnosis [18].

Clinical implementation of computerized electrocardiography occurred in the early 1970s and has continued to develop at a rapid rate. Based on the consensus standards the American College of Cardiology (ACC)/American Heart Association (AHA) requires 500 supervised ECG readings during the initial training period and 100 yearly to maintain competency in ECG reading skills [19]. The computer-assisted ECG reading is of tremendous help for noncardiologists because they do not interpret sufficient ECGs readings. In selected cases they can get immediate help from cardiologists in interpreting the ECG. This is unfortunately not feasible on every ECG obtained routinely in medical and surgical units or in the ED. For emergency physicians ECG aids in making disposition of patients with chest pain and other cardiac complaints. Based on the ECG finding a patient can be admitted or discharged for outpatient follow-up.

Several studies have looked into patients' outcome based on ECG reading by ED physicians compared with cardiologist and computer-assisted ECG readings. Snyder et al. found significant discordance in the ECG interpretation accuracy between the ED physician and the computer-generated reading [20]. McCarthy et al. found that 1.9% of patients were discharged inappropriately of which 25% had missed ST elevations [21]. Westdrop et al. reported in their study that there was only 42% agreement between the ECG reading of the ED physicians and cardiologists with 17.5% interpretation error by the ED physician that was clinically significant [22]. Khun et al. found that 59.2% had agreement with reference standard on major abnormality, but 8.3% had serious interpretation error [23].

A comparison of the different programs has been conducted [24, 25]. Systems processing ECGs have grown from 85 in 1975 to 15,000 over the years, but no consensus was found on which program is more accurate in interpreting ECGs [25]. ECGs of patients in seven common diagnoses were compared between cardiologist and different computer programs. The percentage of ECGs correctly classified by the computer programs was lower than the cardiologists [24].

Despite the sophistication and growing number of computer systems for ECG interpretation and diagnosis, can physicians solely rely on the automated ECG interpretation? The answer to this question is not simple. For instance recently detected new pathologies will not be fed into the computer system before the machines are in use. Therefore, physicians should interpret ECGs despite normal readings by the computer. For instance, Wellens and his group discovered that 75% of patients who had biphasic T wave in leads V2 and V3 (Wellens' sign) on their initial ECG and who were treated medically developed extensive anterior wall infarction within few days [1]. These observations led to a subsequent study by the same group who found critical proximal LAD lesions on coronary angiography [26]. It is recommended that patients with Wellens' sign should not undergo ergometric stress test as this can precipitate extensive MI and could lead to death [1, 26, 27]. The best management for patients with Wellens' sign is coronary angiography followed by PCI or CABG based on their angiographic findings [28, 29].

T wave changes in lead aVL might be considered nonsignificant by most physicians; however, a limited number of studies have shown the importance of T wave changes in recognition of right ventricular involvement in inferior wall MI [30, 31] and sign of mid-LAD lesion [10, 11, 32]. Farhan et al. found 14.1% of the ECGs they reviewed had TWI in lead aVL. In their study they identify isolated T wave inversion to be the only ECG variable significantly predicting mid-segment LAD lesion [32]. They demonstrated that TWI in lead aVL correlated with significant mid-LAD lesion. All ECGs with the isolated TWI in aVL were read as normal by the referring physicians [32]. ST segment changes in lead aVL are also considered as a sensitive marker for early inferior wall MI (early reciprocal change) [8]. Studies have indicated that ST segment or T wave abnormality in specific leads can signify a significant lesion of a specific coronary artery. As shown by Wellens' group there is a higher morbidity from a LAD lesion due to the involvement of larger areas of the myocardium. The accumulating evidence with regard to TWI in aVL indicates that this specific ECG finding should not be considered nonspecific and the diagnosis should not be missed as it potentially leads to significant morbidity and mortality. Unfortunately, T wave changes in lead aVL have not been emphasized and are not well recognized across all specialties.

Our survey indicated that only 25.1% of physicians identified the isolated TWI in lead aVL. The computer read the ECG as normal. The majority of physicians did not recognize the abnormality. In addition, the abnormality was not recognized uniformly across all specialties. Emergency physicians were better than other specialties in recognizing the TWI in lead aVL. A comparison between EM physicians and cardiologists might be a better comparison.

Our patient presented with symptoms suggestive of ACS. He had a recent nuclear stress test that was negative and normal ECG. He presented to the ED with symptoms suggesting ACS and significant changes on his ECG. He had two changes that signified a lesion in the LAD. The first one is Wellens' sign that signifies a proximal LAD lesion and T wave inversion in lead aVL signifying a mid-LAD lesion. The T wave changes in lead aVL suggested the following: (1) an early sign for an acute MI or a sign for a mid-LAD lesion; (2) the presence of Wellens' sign suggesting the possibility of a proximal LAD lesion. Interestingly, on literature review, in 22% of patients with biphasic T waves in leads V2 and V3 the lesions identified on angiogram were in the mid-segment of the LAD [33]. Both findings lead to the decision to take the patient for coronary angiography. Angiography revealed a 99% mid-segment LAD lesion that would go along with the TWI in lead aVL.

Dynamic ECG changes are helpful in detecting ongoing myocardial injury. Our patient's ECG showed the dynamic change from T wave flattening to mild TWI and then prominent TWI in lead aVL. TWI in lead aVL may be an early reciprocal change for acute MI and may be a sign for a mid-segment LAD lesion leading to the initiation of early therapeutic interventions. Left anterior descending artery lesion LAD supplies a larger portion of the heart and since the myocardium at risk from a LAD lesion is large that may lead to significant morbidity or mortality; therefore, it is of paramount importance to recognize subtle changes on ECG such as T wave inversion in lead aVL or Wellens' sign to make an early diagnosis and initiate appropriate treatment in a timely fashion.

The results of a recent study by our group indicated that TWI was associated with mid-LAD lesion with a sensitivity of 87.8 and positive predictive value of 81% for significant mid-LAD lesion (50% and above on coronary angiography) when evaluating angiograms for ST elevation MIs (STEMI). Patients who underwent coronary angiography for other reasons demonstrated a sensitivity of 65.2%, PPV of 83.3%, and specificity of 66.7 for significant mid-LAD lesion (70% and above on coronary angiography) [10].

Industries continue to improves the accuracy of the computer-assisted ECG reading, but as newer findings accumulate older computer programs lag behind and do not recognize findings such as Brugada syndrome, Wellens' sign, or isolated TWI in lead aVL. Therefore, physicians should not solely rely on the computer reading and should look for subtle, yet significant, findings independently of the computer reading. Health care providers should be aware of these subtle findings and it should be emphasized in their training.

One important point that needs to be emphasized is that T wave inversion in lead aVL can be a normal finding. The frequency of its presence in normal population is not known, but frequency is ranging from 10 to 20% in normal Caucasian population in Scotland [34]. In addition, the magnitude of T wave inversion may play a role to be a pathologic finding. Qualitative and quantitative description of T wave inversion have been described and may help differentiate pathological T wave inversions [35–37]. One example is the Pardee T wave in which a T wave inversion in any precordial leads of at least 0.06 mV is considered to predict changes from ischemic heart disease [38]. Other factors such as ventricular hypertrophy or bundle branch block that possibly change the polarity of T wave should be taken into consideration. The above-mentioned factors are limitations to the use of TWI in lead aVL as a sole criterion to predict CAD. The sole presence of isolated T wave inversion may not automatically predict CAD and may represent a normal finding, but the whole clinical picture, the presence of risk factors as well as patients presenting symptoms in combination with a T wave inversion of specific magnitude, may suggest ischemic heart disease.

*Limitations*. The study is limited by small sample size. In addition, the proportion of physicians in each specialty was not equally represented. This may change the final result if an equal number of physicians were included in each group. Finally, the level of training regarding ECG reading in each department and at the different sites is not obvious. Some programs may emphasize ECG reading and the residents get intensive ECG review whereas others may not have equivalent education.

## 6. Conclusion

The computer is a helpful instrument in accurately identifying common pathologies on ECG; however, certain conditions, especially newer findings, are missed by the computer. Physicians need to carefully evaluate ECGs and should not rely on the computer readings. Our study demonstrated that significant number of physicians of all specialties missed the diagnosis. Health care providers should remain alert to new ECG findings and this should be emphasized in their training.

## Figures and Tables

**Table 1:** The number of physicians who did and did not identify the isolated TWI in lead aVL by specialty and training level.

Groups	Identified TW in lead aVL	Not identified TW in lead aVL
EM1	2	3
EM2	8	3
EM3	2	10
EM4	1	4
EMA	2	8

FP1	5	9
FP2	1	8
FP3	4	8
FPA	1	

IM1	6	30
IM2	7	21
IM3	5	16
IM4		1
IMA	3	2

S1		9
S2		3
S3	1	4
S4		2
S5		2
SA		

EM: Emergency Medicine; PF: Family Practice; IM: Internal Medicine; S: Surgery.

## References

[1] de Zwaan C., Bar F. W. H. M., Wellens H. J. J. (1982). Characteristic electrocardiographic pattern indicating a critical stenosis high in left anterior descending coronary artery in patients admitted because of impending myocardial infarction. *The American Heart Journal*.

[2] Jayroe J. B., Spodick D. H., Nikus K. (2009). Differentiating ST elevation myocardial infarction and nonischemic causes of ST elevation by analyzing the presenting electrocardiogram. *American Journal of Cardiology*.

[3] Brady W. J., Perron A. D., Chan T. (2001). Electrocardiographic ST-segment elevation: correct identification of acute myocardial infarction AMI and non-AMI syndromes by emergency physicians. *Academic Emergency Medicine*.

[4] Brady W. J., Perron A. D., Ullman E. A. (2002). Electrocardiographic ST segment elevation: a comparison of AMI and non-AMI ECG syndromes. *American Journal of Emergency Medicine*.

[5] Kracoff O. H., Adelman A. G., Oettinger M. (1993). Reciprocal changes as the presenting electrocardiographic manifestation of acute myocardial ischemia. *The American Journal of Cardiology*.

[6] Kracoff O. H., Adelman A. G., Marquis J.-F., Caspi A., Aldridge H. E., Schwartz L. (1990). Twelve-lead electrocardiogram recording during percutaneous transluminal coronary angioplasty. Analysis of reciprocal changes. *Journal of Electrocardiology*.

[7] Glancy D. L., Doghmi W. (2001). Use of indicative and reciprocal electrocardiographic changes to help localize the site of coronary occlusion. *Proceedings (Baylor University. Medical Center)*.

[8] Birnbaum Y., Sclarovsky S., Mager A., Strasberg B., Rechavia E. (1993). ST segment depression in aVL: a sensitive marker for acute inferior myocardial infarction. *European Heart Journal*.

[9] Farhan H. L., Hassan K. S., Al-Belushi A., Sallam M., Al-Zakwani I. (2010). Diagnostic value of electrocardiographic T wave inversion in lead aVL in diagnosing coronary artery disease in patients with chronic stable angina. *Oman Medical Journal*.

[10] Hassen G. W., Costea A., Smith T. (2014). The neglected lead on electrocardiogram: T wave inversion in lead aVL, nonspecific finding or a sign for left anterior descending artery lesion?. *The Journal of Emergency Medicine*.

[11] Hassen G. W., Talebi S., Fernaine G., Kalantari H. (2014). Lead aVL on electrocardiogram: emerging as important lead in early diagnosis of myocardial infarction?. *The American Journal of Emergency Medicine*.

[12] Staples L. F., Gustafson J. E., Balm G. J., Tate W. A. (1966). Computer interpretation of electrocardiograms. *American Heart Journal*.

[13] Caceres C. A., Steinberg C. A., Abraham S. (1962). Computer extraction of electrocardiographic parameters. *Circulation*.

[14] Pipberger H. V., Arms R. J., Stallmann F. W. (1961). Automatic screening of normal and abnormal electrocardiograms by means of digital electronic computer. *Proceedings of the Society for Experimental Biology and Medicine*.

[15] Pipberger H. V., Freis E. D., Taback L., Mason H. L. (1960). Preparation of electrocardiographic data for analysis by digital electronic computer. *Circulation*.

[16] Pipberger H. V. (1964). Use of digital computers in analyzing electrocardiographic data. *The Heart Bulletin*.

[17] Stallmann F. W., Pipberger H. V. (1961). Automatic recognition of electrocardiographic waves by digital computer.. *Circulation Research*.

[18] Pipberger H. V., McCaughan D., Littmann D. (1975). Clinical application of a second generation electrocardiographic computer program. *The American Journal of Cardiology*.

[19] Kadish A. H., Buxton A. E., Kennedy H. L. (2001). ACC/AHA clinical competence statement on electrocardiography and ambulatory electrocardiography. A report of the ACC/AHA/ACP-ASIM Task Force on Clinical Competence (ACC/AHA Committee to Develop a Clinical Competence Statement on Electrocardiography and Ambulatory Electrocardiography). *Journal of the American College of Cardiology*.

[20] Snyder C. S., Fenrich A. L., Friedman R. A., Macias C., O'Reilly K., Kertesz N. J. (2003). The emergency department versus the computer: which is the better electrocardiographer?. *Pediatric Cardiology*.

[21] McCarthy B. D., Beshansky J. R., D'Agostino R. B., Selker H. P. (1993). Missed diagnoses of acute myocardial infarction in the emergency department: results from a multicenter study. *Annals of Emergency Medicine*.

[22] Westdorp E. J., Gratton M. C., Watson W. A. (1992). Emergency department interpretation of electrocardiograms. *Annals of Emergency Medicine*.

[23] Kuhn M., Morgan M. T., Hoffman J. R. (1992). Quality assurance in the emergency department: evaluation of the ECG review process. *Annals of Emergency Medicine*.

[24] Willems J. L., Abreu-Lima C., Arnaud P. (1991). The diagnostic performance of computer programs for the interpretation of electrocardiograms. *The New England Journal of Medicine*.

[25] Drazen E., Mann N., Borun R., Laks M., Bersen A. (1988). Survey of computer-assisted electrocardiography in the United States. *Journal of Electrocardiology*.

[26] de Zwaan C., Bar F. W., Janssen J. H. A. (1989). Angiographic and clinical characteristics of patients with unstable angina showing an ECG pattern indicating critical narrowing of the proximal LAD coronary artery. *American Heart Journal*.

[27] Tandy T. K., Bottomy D. P., Lewis J. G. (1999). Wellen's syndrome. *Annals of Emergency Medicine*.

[28] Boden W. E., Bough E. W., Benham I., Shulman R. S. (1983). Unstable angina with episodic ST segment elevation and minimal creatine kinase release culminating in extensive, recurrent infarction. *Journal of the American College of Cardiology*.

[29] Mead N. E., O'Keefe K. P. (2009). Wellen's syndrome: an ominous EKG pattern. *Journal of Emergencies, Trauma and Shock*.

[30] Rashduni D. L., Tannenbaum A. K. (2003). Utility of ST segment depression in lead AVL in the diagnosis of right ventricular infarction. *New Jersey Medicine*.

[31] Turhan H., Yilmaz M. B., Yetkin E. (2003). Diagnostic value of aVL derivation for right ventricular involvement in patients with acute inferior myocardial infarction. *Annals of Noninvasive Electrocardiology*.

[32] Hassen G. W., Kalantari H. (2012). Diplopia from subacute bilateral subdural hematoma after spinal anesthesia. *Western Journal of Emergency Medicine*.

[33] Akhtar P., Rizvi S. N. H., Tahir F., Saleem D., Mulla J., Saghir T. (2012). Angiocardiographic findings in patients with biphasic T-wave inversion in precordial leads. *Journal of the Pakistan Medical Association*.

[34] Macfarlane P. W., van Oosterom A., Janse M., Kligfield P., Camm J., Pahlm O. (2012). *Specialized Aspects of ECG*.

[35] Chikamori T., Doi Y. L., Furuno T., Yonezawa Y., Ozawa T. (1992). Diagnostic significance of deep T-wave inversion induced by exercise testing in patients with suspected coronary artery disease. *American Journal of Cardiology*.

[36] Rautaharju P. M., Surawicz B., Gettes L. S. (2009). AHA/ACCF/HRS recommendations for the standardization and interpretation of the electrocardiogram: part IV: the ST segment, T and U waves, and the QT interval: a scientific statement from the American Heart Association Electrocardiography and Arrhythmias Committee, Council on Clinical Cardiology; the American College of Cardiology Foundation; and the Heart Rhythm Society. Endorsed by the International Society for Computerized Electrocardiology. *Journal of the American College of Cardiology*.

[37] Hanna E. B., Glancy D. L. (2011). ST-segment depression and T-wave inversion: classification, differential diagnosis, and caveats. *Cleveland Clinic Journal of Medicine*.

[38] Kraft M., French W. J., Laks M. M. (1991). Use of the computer to detect the pardee T wave. Frequent marker of coronary artery disease. *Journal of Electrocardiology*.

